# Graphene-Based Nanomaterials: From Production to Integration With Modern Tools in Neuroscience

**DOI:** 10.3389/fnsys.2019.00026

**Published:** 2019-07-16

**Authors:** Kristina E. Kitko, Qi Zhang

**Affiliations:** ^1^Program in Interdisciplinary Materials Science, Vanderbilt University, Nashville, TN, United States; ^2^Department of Pharmacology, Vanderbilt University, Nashville, TN, United States; ^3^The Brain Institute, Florida Atlantic University, Jupiter, FL, United States

**Keywords:** graphene, graphene oxide, neuron, plasma membrane, cytotoxicity, synapse, neurotransmission

## Abstract

Graphene, a two-dimensional carbon crystal, has emerged as a promising material for sensing and modulating neuronal activity *in vitro* and *in vivo*. In this review, we provide a primer for how manufacturing processes to produce graphene and graphene oxide result in materials properties that may be tailored for a variety of applications. We further discuss how graphene may be composited with other bio-compatible materials of interest to make novel hybrid complexes with desired characteristics for bio-interfacing. We then highlight graphene’s ever-widen utility and unique properties that may in the future be multiplexed for cross-modal modulation or interrogation of neuronal network. As the biological effects of graphene are still an area of active investigation, we discuss recent development, with special focus on how surface coatings and surface properties of graphene are relevant to its biological effects. We discuss studies conducted in both non-murine and murine systems, and emphasize the preclinical aspect of graphene’s potential without undermining its tangible clinical implementation.

## Introduction

The promises of nanomedicine are extensive. Graphene (Gr), the first true two-dimensional material to exist in isolation, is the type of new nanomaterial that results in interest for novel biomedical applications. From Michael Chrichton’s tragic protagonist in *The Terminal Man* to the recent growth in start-up companies seeking to transfer consciousness, the fictive present and future call to mind visions of devices that enable neural interfacing and control. Although these ideas may create questions as to ethics for neuroscience in the future, the current state-of-the-art for implanted devices is far more limited in scope. Progress in brain-computer interfaces holds great promise for patients following stroke (Ramos-Murguialday et al., 2013), to control prosthetic limbs (Hochberg et al., 2006; Donoghue et al., 2007), with the motor degeneration characteristic of Parkinson’s disease (Little et al., 2013), and for a variety of other disorders and diseases (Chaudhary et al., 2016). Gr may be poised for incorporation into such devices. As the presence of Gr becomes more widespread and commonplace across the biomedical sciences, the relatively larger body of work detailing the biological effects of carbon nanotubes may serve as a template guiding the utility of Gr for biological applications (Kostarelos et al., 2009).

Gr-based materials for interfacing with the peripheral nervous system have been reviewed elsewhere (Domínguez-Bajo et al., 2017; Bramini et al., 2018). We instead focus on new directions for application to the central nervous system. This review is limited to preclinical applications, although Gr and Gr-based devices may someday advance to clinical implementation. We begin with an overview of Gr manufacturing advances, applications to hybrid materials systems as well as drug delivery strategies. This is followed by an overview of work performed with non-murine models. Finally, the interaction of Gr with murine neural systems, both *in vivo* and *in vitro* is examined. Despite a sizable body of work, to date, there remain many unresolved questions as to cytotoxicity and the mechanisms underlying the Gr-cellular interaction that must be addressed moving forward.

## Manufacturing Processes

**Figure 1:**
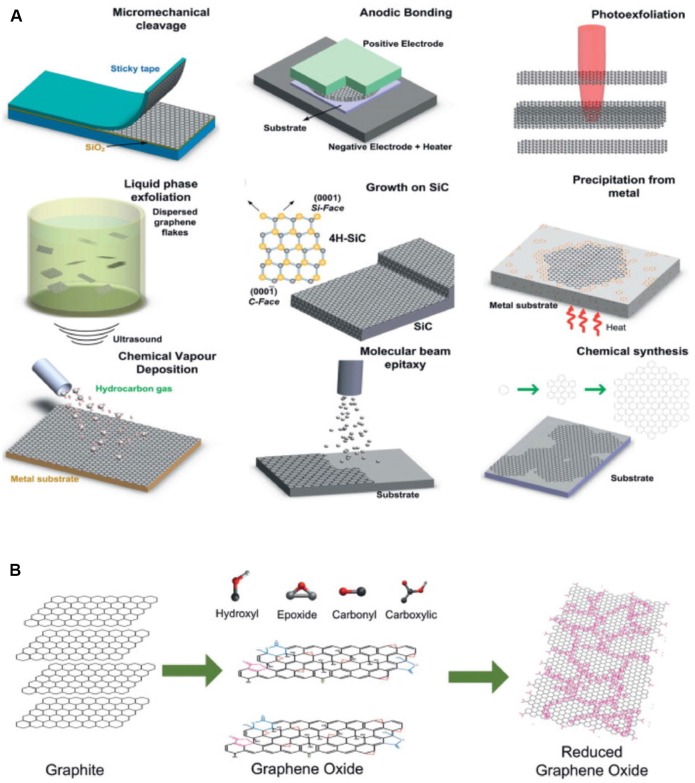
Production of Graphene/Gr oxide (Gr/GO). **(A)** Gr production methods and **(B)** GO production and reduction methods. Modified with permission from Bonaccorso et al. (2012).

2D graphite was long believed to be relegated to the realm of theoreticians and condensed matter physicists, as the thermodynamic stability of such crystals was believed to be prohibitive for their existence (Geim and Novoselov, 2007). 2D graphite—or “graphene”—was first isolated through mechanical exfoliation (Novoselov et al., 2004, 2005). These small sheets provided the ability to study transport in this new class of material (Novoselov et al., 2004), but the small size of the sheets (<10 μm) necessitated the development of alternative approaches that would produce Gr in sizes large enough for practical transistor-based applications. Of note, for high quality single and few layer Gr sheets, mechanical exfoliation remains the process of choice for transport measurements to date. However, an array of production methods are now enabling production of high-quality Gr in ever higher qualities and ever larger areas (Figure 1).

The development of chemical-vapor deposition as a manufacturing strategy (Li et al., 2009a; Reina et al., 2009) allows the production of Gr with higher area coverage than was previously possible with exfoliation-based methods. To date, the majority of devices that demonstrate compatibility for *in vivo* imaging use single or multi-layer Gr produced *via* chemical vapor deposition (Kuzum et al., 2014; Park et al., 2014, 2016, 2018; Du et al., 2015). Indeed, advances in CVD technology have allowed the production of rectangular Gr sheets with a cross-length as long as 30 inches (Bae et al., 2010). Although these large size sheets should be of sufficient size for any neural application, the solution transfer process often results in alteration of the properties of the Gr sheet (Suk et al., 2011, 2013). Thus, continued research into scalable transfer of CVD Gr or alternative processes (Pang et al., 2017) should help generate more reliably responsive devices. Additionally, ensuring that devices are processed in such a way as to remain relatively sterile is a necessary step to consider for long-term interfacing.

As an alternative to the large-scale growth of Gr, it is now possible to produce large quantities of single-layer and multi-layer Gr through bulk exfoliation (Hernandez et al., 2008; Li et al., 2008; Lotya et al., 2009; Shih et al., 2011; Paton et al., 2014). Graphite in a colloidal suspension can be sonicated to yield thin Gr flakes, ranging from single to a few layers. A variety of solvents are compatible with this approach—and more recently it has been demonstrated that Gr and Gr oxide (GO) can be exfoliated directly into biological media (Castagnola et al., 2018). GO flakes produced by this method are more largely monolayer than Gr flakes, however, oxidization comes with costs, for example, reduced carrier mobility. Flakes of Gr produced by exfoliation, while not matching the transport properties of micromechanical isolation, can be considered Gr, as no chemical functionalization is required. Thus, Gr flakes may prove advantageous for applications where the electronic properties are not of primary importance but where large quantities of Gr are desirable. To be noted, long-term stability of Gr flake suspension still requires coating with surfactant as the hydrophobic surface of those flakes readily forcing them to form aggregates (unpublished result).

The ease with which GO can be chemically modified remains of interest for drug delivery and bio-scaffolding applications. A number of different processes have been developed to produce bulk quantities of GO flakes (Brodie, 1860; Hummers and Offeman, 1958), although GO is traditionally produced through the reduction of bulk graphite in the presence of both acids and oxidants (Park and Ruoff, 2009). Reduction of GO yields Gr-like sheets (rGO) with improved electrical conductivity (Stankovich et al., 2007), but the electronic properties of rGO still lag far behind those of pure Gr, even with the numerous efforts that have been made *via* modifying annealing processes to improve the figures of merit. The inherently lower electrical conductivity and inability to greatly increase it has resulted in less interest to date in the use of GO as an electrode material. Indeed, even chemical reduction of GO to rGO yields room temperature conductivity values three orders of magnitude below that measured for pristine Gr (Gómez-Navarro et al., 2007). Mobility values for GO produced *via* the Hummers method are ~850 cm^2^/(V s), but other methods report a device mobility of around ~1–10 cm^2^/(V s) (Eda et al., 2008; Wang et al., 2008; Su et al., 2009). Importantly, future device design may take into account contact resistance in production, as use of all-carbon transistors improve both electron and hole mobility relative to using gold electrodes (Wang et al., 2010), which may prove a more practical approach to improve device performance. Further attempts to reduce GO to Gr-like sheets *via* chemical (Li et al., 2009b; Moon et al., 2010) or thermal (Becerril et al., 2008; Jung et al., 2009; Barroso-Bujans et al., 2010) processing have improved the fraction of graphitic areas in the structure, but also introduced nanoscale holes and defects that deleteriously affect final performance. Ultimately, for practical application, if devices are to be manufactured at a commercial scale, inconsistencies introduced in such devices will have to be carefully characterized to ensure that recorded signals are representative of neural responses. rGO’s improved device characteristics also come at the cost of a reduction in hydrophilicity (Rourke et al., 2011), and surface properties remain an important consideration for bio-interfacing applications. It was recently demonstrated that solution-exfoliated GO flakes could be processed to recover properties more resembling those of CVD-produced Gr. Reduction of the concentration of in-plane oxygen *via* 1–2 s microwave pulses produced 2D and G peaks closer to those of Gr and greatly increased electron and hole carrier mobility to ~1,000 cm^2^/(V s) in a FET (Voiry et al., 2016). Thus, the ability to modify in-plane oxygen concentration to improve electronic properties may open new doors to the use of GO and GO-based materials for sensor applications.

Similar to applications for the photoluminescence of CNTs (Welsher et al., 2008), the photoluminescence of GO may be useful in for optical readout and/or in combination with drug delivery. The photoluminescence of GO, which arises from bond disorder throughout the structure, which induces energy gaps (Cao et al., 2013), is to some extent a tunable property, depending on the oxidization state (Luo et al., 2009). This stands in contrast to the lack of photoluminescent emission observed for defect-free Gr. GO luminescence is broadband in as-prepared samples, with a wide peak across the visible spectrum (Luo et al., 2009; Qian et al., 2012). PEGylated GO sheets exhibit intrinsic near-infrared red Hernandez et al., 2008 photoluminescence (Sun et al., 2008), a property of great interest for *in vivo* imaging applications due to the enhanced light penetration in this wavelength range. It has been more recently demonstrated that GO also exhibits photoluminescence under both two-photon and three-photon excitation (Qian et al., 2012), in addition to the broad photoluminescence in the visible range. By exploiting the ability of GO to undergo a two-photon process, Qian et al. (2012) imaged PEGylated-GO nanoparticles in a skull-removed whole brain to a depth of 300 μm. Growth in the commercial availability of three-photon sources may lead to studies at even greater depths within the intact rodent brain. As new advances in genetic engineering and microscopy enable deeper and faster cellular resolution imaging in head fixed or freely moving specimens, it is likely GO/Gr-based imaging will also move toward applications compatible with this type of experiment. Although the broadband nature of the emission from GO may be somewhat of a limiting factor for multiplexing multiphoton imaging processes, advances in hyperspectral detection and fast fluorescence lifetime detection may help to make GO of greater utility for *in vivo* brain imaging.

## Graphene/Polymer Composite Materials and Applications

Polymer electronics remain of great interest to ultimately offer an alternative to traditional materials to minimize mechanical mismatch between cells/tissue and the recording probe (for a more in-depth discussion see Rivnay et al., 2017). Elastic modulus mismatch between brain tissue and recording device leads to increased tissue damage both upon insertion and during chronic interface (Polikov et al., 2005), as the elastic moduli of brain tissue (~150 kPa) and an implanted electrode (~150 GPa for silicon) differ by six orders of magnitude. Thus, much current research has focused on the ability to better match the modulus of electrodes to that of brain tissue (for a more in-depth review on interfacing tissue with electrodes, see Fattahi et al., 2014). Gr electrodes have been of interest for such designs, as it can be incorporated into flexible electronics (Fiori et al., 2014); and for applications in the brain, where techniques like optogenetics and calcium imaging require optical access to brain regions of interest, the large degree of optical transparency of single or few-layer Gr (FLG) may be uniquely advantageous.

## PEDOT

Poly-3, 4-ethylene dioxythiophene (PEDOT) is an electroconductive polymer produced from 3, 4-ethylene dioxythiophene monomers. Polymerization results in a positively-charged backbone, whereby negatively charged materials can then be incorporated to balance charge. Neurons embedded in PEDOT matrices remain viable for around 1 week (Richardson-Burns et al., 2007) and neurons grown on PEDOT-based substrates show unaltered electrophysiological characteristics (membrane potential, membrane capacitance, input resistance) after 21 DIV (Cellot et al., 2016). Although the surface charge of Gr limits its utility in PEDOT-based composites without further surface modification, the negative surface charge of GO may be better adaptable to such composites. PEDOT/GO composites have been used as electrode coatings to improve sensitivity and decrease the lower detection limit of dopamine in fast-scan cyclic voltammetry (Taylor et al., 2017), a widely-used technique for measuring dopamine release in rodents *in vivo* (Robinson et al., 2003). Carbon nanotube-PEDOT composites have also been demonstrated to perform well in interfacing applications (Jan et al., 2009; Luo et al., 2011). PEDOT-coated microelectrode arrays show good performance characteristics and lowered impedance relative to iridium oxide (IrOx), suggesting a potential for long-term neural interfacing applications (Wilks et al., 2009). As both GO and CNT incorporation into PEDOT-based composites has improved overall performance, future work may seek to fabricate composite polymer electrodes.

### Chitosan

Chitosan composites have been demonstrated both for GO (Yang et al., 2010; Bao et al., 2011) and for rGO (Fang et al., 2010). It can be produced in relatively abundant quantities from the deacetylation of chitin. Like with many polymers, applications may be limited by the low mechanical strength of the material. GO as a nanofiller is one route to achieve enhanced mechanical properties. Chitosan-GO nanocomposites can be assembled in a manner of ways. pH-responsive functionality is possible with chitosan (Yi et al., 2005); increased pH leads to amine deprotonation, decharging of the polymer, and ultimately insolubility. Interestingly, preparations of such suspensions seem to be greatly affected by the preparation method: addition of GO to chitosan yields a uniform suspension while addition of chitosan to GO yields agglomerations (Fang et al., 2010). This is due to the way that excess GO will create bridges between sheets *via* multiple attachment points on the polymer chains. Reducing GO allows chitosan attachment by zwitterionic interactions and hydrogen bonding between the remaining oxygen groups of the rGO and the amino and hydroxyl groups of chitosan. The reversibility of the molecular chain interactions with GO sheets between different pH values may provide an opportunity to modulate Gr-based composite materials within different cellular compartments. This could potentially allow for pH-based assembly strategies in acidic intracellular compartments (e.g., late endosomes, lysosomes), where chitosan would stabilize GO composites relative to higher pH extracellular spaces.

### Hydrogels

The use of Gr for regenerative approaches has been reviewed previously (Ding et al., 2015), however the pace of new methodologies in neuroscience has opened new directions for scaffolding technologies, with a particular resurgence in hydrogel-based techniques for connectome applications (Chung et al., 2013; Chen et al., 2015). Gr/GO and other nanomaterials may be of interest for cleared or expanded tissue applications where added structural stability is desirable. Composite scaffolds for regenerative medicine remain and area of great interest. Hydrogels can be chemically tuned to impart different surface properties, for example, to modify surface charge or conductivity, before functionalization to GO (Liu X. et al., 2017). Biomolecules such as DNA can also be incorporated *via* stacking interactions (Xu et al., 2010), enabling payload delivery within the hydrogel matrix. This is one possible route to achieve more biologically realistic synthetic minimal brain circuits, which to date have largely been limited in structure to two-dimensional culture systems or proteins or liposomes (Adamala et al., 2017). Overall mechanical strength can be tailored by the degree to which Gr/GO is incorporated, with mechanical strength being inversely correlated to the amount of swelling in the composite. Local delivery of polymerized materials may someday be a route to a new form of tissue scaffolding *in vivo*. In such applications, Gr and Gr-based materials may play a multifunctional role, both as structural support and as part of a stimulation or recording device.

### Graphene in “Stretchable” Electronics Applications

Advances in both computational and analytical models have recently begun to enable the fabrication of nanoscale semiconductor materials that will tolerate relatively large amounts of strain (Su et al., 2017; Yu et al., 2017), advances in the manufacturing of Gr may allow similar structures to be produced (Wang et al., 2017). Although measurements of second-order stiffness in graphene have yielded in-plane stiffness values of ~340 N/m (Lee et al., 2008), crumpling from static wrinkling in free-standing Gr at biologically relevant temperatures effectively reduces this value (Nicholl et al., 2015). p-type doping of Gr may be one way forward for flexible Gr electrodes, as it decreases sheet resistance and increases the effective work function (Han et al., 2016). Multilayer-based approaches using Gr may also improve stretchability performance through strain relaxation (Won et al., 2014). In fact, the addition of Gr “nanoscrolls” between layers in transparent transistors showed improved performance under strain relative to monolayer Gr (Liu N. et al., 2017); and multilayer composite or flexible devices must be designed with consideration to the properties of Gr/GO that are being utilized. For example, PEDOT electrodes with sufficient recording capability where Gr may be incorporated to increase overall mechanical strength may have different design criteria from applications where Gr acts as an electrode material.

## Drug Delivery Applications and Conjugation Strategies

### Chemical Modifications for Drug Delivery Applications

Gr/GO have been most widely demonstrated for cancer-related drug delivery applications (Liu et al., 2011; Liu C.-C. et al., 2017), however, the chemical and surface modifications used to enable loading and release may also be used to enable new applications in the brain. The ability to harness hydrophobic interactions and π—π stacking to deliver aromatic, hydrophobic compounds may extend the utility of Gr for brain-specific drug delivery beyond simply proof-of-concept. For example, polyethylene glycol modification (PEGylation) of GO results in excellent solution stability (Liu et al., 2008). Alternative strategies also include Polyamidoamine (PAMAM) functionalization of both GO (Gu et al., 2017) and Gr (Quintana et al., 2011) and hyperbranched polyglycerol (hPG; Tu et al., 2017). Amide linkage between GO and chitosan yields sheets that are relatively stable in cell culture media for up to 48 h (Bao et al., 2011), an example of a myriad of alternative approaches to stabilize Gr/GO in aqueous solutions. Dextran can also be used to increase the hydrophilicity of GO *via* amine modification and EDC coupling chemistry (Zhang et al., 2011). GO functionalized with cyclodextrin molecules, again *via* π—π adsorption, reduced to Gr sheets in an ammonia solution also serves as an effective peptide carrier (Dong et al., 2013). Thus, the versatility of π—π adsorption onto Gr/GO surfaces allows for a wide scope of possible molecular delivery types.

Chemical reaction methods can also be selected to control the location of functional groups onto Gr sheets. 1,3-dipolar cycloaddition results in conjugation within the large central area of the sheets, whereas amide concentration reactions concentrate conjugates to the edges of Gr sheets (Quintana et al., 2011). Azide modified dopamine has been used for simultaneous capping and reduction of GO (Kaminska et al., 2012), where the aromatic structure of the dopamine molecule likely interacts *via* π—π stacking on the surface. As many monoamines contain aromatic groups, molecules such as serotonin, cathecholamine, and epinephrine may find utility as stabilizers for GO while also acting to alter neural function. Although click chemistry opens new doors to functionalization strategies for Gr/GO, approaches that utilize a copper catalyst elicit concern regarding toxicity to living tissue (Baskin et al., 2007).

Interestingly, the overall surface charge of a GO sheet was shown to play a role in the effectiveness of intracellular drug delivery. Positively-charged aminolated surfaces were shown to be more effective at releasing Doxorubicin (DOX) in intracellular compartments than negatively charged sulfonated surfaces (Tu et al., 2017). Given the relative ease of modifying the surface charge of GO, this may be a new avenue to site-specific intracellular drug release. The different surface characteristics across a Gr sheet may also be a useful strategy for orthogonal delivery of different classes of compound: the negatively charged surface regions may better adsorb positively charged molecules while the outer edges, decorated with carboxyl groups, can, for example, be modified with zwitterionic lipid vesicles (Wang et al., 2013).

### Payload Delivery

The delivery of various forms of genetic payload has been demonstrated as a possible application for Gr/GO-based materials, however, to date, low transduction efficiencies limit the utility of Gr in comparison to traditional methods for genetic delivery. For example, chitosan-stabilized GO sheets had a lower transfection efficiency for luciferase transduction into HeLa cells (Bao et al., 2011) compared to traditional methods. Polymer-based assemblies are some of the most widely used nanomaterial strategies for transduction, and relative to polymer-based approaches, GO used to attach plasmid DNA and PEI shows improved transfection efficiency (Feng et al., 2011). Gr can also bind ssDNA, although cannot bind dsDNA to the same extent. This property has been exploited to deliver hairpin-shaped DNA into cells, which will be unloaded upon interaction with an mRNA target (Lu et al., 2010). GO has also been employed as a delivery vehicle for aptamers, delivering an ATP-binding aptamer (Wang et al., 2010) to cells. Gr/GO may ultimately be most advantageous for applications where simultaneous delivery of both genetic payloads and pharmacological compounds is desirable. For example, PAMAM functionalized GO was demonstrated as a vehicle for both DOX and shRNA (Gu et al., 2017) delivery.

### Photothermal Therapy

Reduced GO (rGO) has been exploited for its photothermal properties for drug delivery. Chitosan/rGO composites were shown to deliver drug payloads on a timescale of minutes; addition of rGO to Chitosan acts to increase the photothermal absorption of the composite with respect to chitosan alone (Matteini et al., 2014). Here, DOX delivery to HeLa cells was increased with short-pulse laser illumination. DOX has also been loaded onto GO for photothermal delivery in a glioma-bearing rat model (Liu et al., 2013; Dong et al., 2016). Laser irradiation results in local surface heating, ultimately leading to drug release. DOX release was also demonstrated to be effective on gliomas when loaded on PEGylated silica-coated Gr sheets (Wang et al., 2013). Carboxy-modified GO was covalently linked to Thioflavin S, which made it selectively attached to amyloid-β fibrils (Li et al., 2012), a potential avenue toward the photothermal dissociation of Aβ fibrils and demonstrating the potential of Gr/GO-based materials for therapeutic application to Alzheimer’s disease. Hydrazine reduction of GO at elevated temperatures increases NIR absorbance by >6-fold relative to unreduced GO (Robinson et al., 2011), a function of the restoration of π conjugation. In the most widely used state, largely due to the relative ease of production and low cost, Gr flakes exist as a semimetal with zero bandgap. More recently, the discovery that a “nanomesh” structure can open up a bandgap in Gr (Akhavan, 2010; Bai et al., 2010) can be used to tune photothermal absorption properties. PEGylated rGO nanomesh suspension showed a much steeper temperature increase over time for NIR irradiation heating than PEGylated rGO (Akhavan and Ghaderi, 2013). Although CNTs have been more widely used for photothermal therapy to date, the superior response of Gr (Markovic et al., 2011) may lead to increased focus in this direction.

Laser irradiation with NIR light enables a relatively high degree of spatial precision. However, for *in vivo* applications, the ability to control release will ultimately be limited by the ability to deliver light within the brain. As such, NIR will be a useful tool for fundamental studies, as differential effects between even superficial sub-regions within the brain are still not well understood. Alternative triggering methods may be better suited to study where pharmaceutical effects are elicited in deep brain regions. Electrical, magnetic, or even acoustic-based triggered release would allow such control in deeper brain regions. Layer-by-layer assembly approaches utilize protein adsorption onto substrates and subsequent capping with modified GO in either a sheet (Hong et al., 2012) or a capsule (Kurapati and Raichur, 2012) format. Additional layers can be stacked together to control overall release time (Hong et al., 2012). Passive release may be sufficient for the delivery of certain drug classes, but active release allows more precise control of treatment dose received. PAE (Choi et al., 2015) or PPy (Weaver et al., 2014) films can incorporate GO, resulting in a more conductive polymer matrix, whereby electrical stimulation is applied and elicits drug release. Modification of the number of GO layers and the overall areal size of the GO sheets also alters the total drug loading capability (Weaver et al., 2014); smaller and fewer layered sheets have increased surface area for adsorption relative to more multilayered stacks. Photothermal irradiation has also been utilized to target delivery to cytosolic locations. Although Gr/GO sheets can insert into membranes directly, small sized and few-layered sheets will also be taken up into the cell through endocytic processes. Ultimately these sheets will then be trafficked to endosomal compartments. rGO sheets have been used to help payload escape this fate by application of NIR irradiation to induce endosomal disruption (Kim et al., 2013).

### Magnetic Applications

The ability to modify the properties of Gr/GO, to confer magnetic sensitivity for example, will extend the utility of its applications. The presence of fluorine in the GO basal plane can induce paramagnetic centers, making fluorinated-GO compatible with MRI applications (Romero-Aburto et al., 2013). Magnetic nanoparticles such as iron oxide (Fe_3_O_4_) can also be loaded on the surface of GO, conferring sufficient contrast enhancement to enable MRI (Yang et al., 2013). This does not disrupt the ability of Gr/GO to act as a drug delivery vehicle, further extending its utility.

## Toxicity in Murine Systems

The extent to which Gr/GO become practically applicable to neuroscience will in part be determined following a systematic understanding of long-term toxicity. As many paths toward clinical application begin with pre-clinical testing in murine models, understanding the biological tolerance of rats and mice to Gr/GO represents an important first step. Here, we focus on toxicity specifically relevant to neuronal and brain-wide function, for discussion of overall, environmental, or antimicrobial toxicity, which have been widely reviewed, see elsewhere (Seabra et al., 2014).

Various studies have also focused on the interaction between Gr and the cell membrane in either *in vitro* culture systems (Kitko et al., 2018) or lipid bilayer preparations. Using a two-dimensional Langmuir-Blodgett approach, it has been suggested that the hydrophobic tail of lipids does not play a role in any bilayer interactions, but a positively charged head group would favor interactions with the carboxy-containing regions of GO (Li et al., 2013) and minimal interactions would occur between neutrally or negatively charged lipids. The size of the Gr/GO flakes is also a determining factor in the bilayer response. Flakes of GO that are large relative to the size of an artificial liposome cause rupture of the bilayer attached to a substrate surface (Frost et al., 2012). Addition of GO to supported lipid bilayers (SLBs) composed of DPPC/DOPC causes detachment of bilayer regions (Lei et al., 2014), but as this was a function of relatively high levels of calcium used in SLB preparation, may not be viewed as representative of *in vivo* membrane damage.

The results based on computational modeling generally agree that once inside of a lipid bilayer, either *via* endocytic uptake or by direct membrane penetration, Gr will stably reside between phospholipid tails (Titov et al., 2010; Guo et al., 2013). It is generally agreed that membrane penetration would favor an “edge-in” rather than a “face-in” initial contact.

Although computational models are powerful tools to provide fundamental insights into the forces governing Gr/GO nanomaterial-cellular interactions, these studies are often performed under conditions necessitated by restrictions on the ability to model different membrane components, for example, protein coronas and extracellular-matrix components secreted by neurons are largely not included in such models. It is furthermore prohibitive to model the membrane bilayer with the full complexity of proteins, lipids, and other molecules within a neuronal bilayer. In addition, it is computationally prohibitive to model Gr/GO flakes on the same size scales that are produced for experimental studies. A “large Gr/GO flake” in a computational study may be on the length scale of 5 nm (Li et al., 2013)—whereas for experimental studies the smallest reported average dimensions are on a length scale of >200 nm (Rauti et al., 2016; Castagnola et al., 2018). Thus, it may be difficult to draw direct parallels between toxicity claims from simulations and toxicity claims from experimental results. Study of lipid-membrane specific effects is more efficiently enabled by allowing Gr to penetrate a membrane after addition to biological media. However, many studies, even using small flakes of Gr/GO, deposit the material onto a glass substrate for chronic cellular interface. The membrane interactions here would be very different from Gr/GO located within the lipid bilayer, further complicating arguments as to the membrane effects of Gr/GO; and same caution should be paid to the bare or surfactant-coated Gr as the former will aggregate and result in different physicochemical properties.

GO was demonstrated to be toxic to gram-negative bacteria (Akhavan and Ghaderi, 2010; Tu et al., 2013), yet bacteria containing more complex outer membranes are more resistant to damage. Reduction of GO (rGO) increases susceptibility to membrane damage (Akhavan and Ghaderi, 2010). Akhavan (2010) fabricated nanowalls of GO, which were designed such that there would be a maximal amount of direct contact between bacteria and the sharp edges of the nanomaterial. This represents a condition that would induce mechanical stress on the membrane and indeed can result in membrane damage. Tu et al. (2013) later extended this work both through molecular dynamics simulations and experiments using *E. Coli*. Course-grained molecular dynamic simulations of relatively large FLG sheets suggest that the most hydrophobic edge of Gr near a lipid bilayer will penetrate orthogonal to a bilayer, and then fully embed in a membrane, driven by an attraction between the Gr and lipids within the core (Li et al., 2013). Interestingly, this spontaneous process does not result in membrane destruction, suggesting that the degree of mechanical stress on the membrane plays a role in membrane damage when exposed to Gr or GO. That spontaneous membrane incorporation does cause membrane destruction is in agreement with experimental observations using cultured PC-12 cells, where 24-h exposure to FLG sheets did not increase lactate dehydrogenase activity or increase reactive oxygen species below 100 μg/mL treatment concentrations (Zhang et al., 2010).

Of note, these configurations, where membrane stress is likely a factor in the toxicity of Gr/GO, are different than most studies to date using neuronal cultures, where Gr/GO is more commonly used as a culture substrate. Three different mammalian cell types cultured on rGO, but not GO, for up to 5 days proliferated normally and exhibited less cytotoxicity and more outgrowth than on CNT films (Agarwal et al., 2010). HT-29 cells also displayed increased attachment on GO-coated substrates within 6 h compared to bare glass substrates (Ruiz et al., 2011). Thus, future studies aimed at addressing nanotoxicity would benefit from drawing a distinction between scenarios where mechanical stress may be an additional factor and scenarios where spontaneous membrane incorporation alone is being studied.

Simulations also suggest that the hydrophobicity of Gr/GO plays a role in its interaction with the bilayer and that the surface energy can be modified by the formation of a protein corona on the surface. For example, computational models demonstrate that the presence of a protein corona surrounding the flakes would modify the membrane response to Gr, in which case Gr would orient in parallel to and attach to the outer layer of the lipid bilayer (Li et al., 2013). Experimental studies of protein adsorption on Gr/GO alone have shown that nanoflakes can adsorb 1.6–2× their weight in BSA, largely on the timescale of minutes (Hu et al., 2011; Chong et al., 2015). A recent development, where Gr is exfoliated directly into a serum containing media, has given some insight into the composition of the biological corona formed. Proteomic analysis of these media-exfoliated Gr flakes reveals a variety of proteins and other cellular materials that make up the protein corona formed on Gr (Castagnola et al., 2018): serum albumin, apolipoproteins, and vitronectin are all found on Gr nanoflakes in relatively large abundances. Although the physicochemical interactions, kinetics, and thermodynamic processes that govern the formation and evolution of the corona that forms around nanomaterial surfaces when interfaced with a biological system is still not fully understood, general frameworks have been established as to the governing forces underlying nano-bio interactions. Because the individual environment Gr/GO encounter will vary widely depending upon desired neural application, the exact composition of the corona formed cannot necessarily be described *a priori* or results extended from one biological system to the next. Indeed, the chemical concentration, surface functionalization, degree of crystallinity, and surface roughness, among many properties, all play a role in the composition and evolution of biomaterials that adsorb on a nanomaterial surface (Nel et al., 2009).

The formation of a protein corona on Gr/GO surfaces also differs between bacterial cultures and what would be observed in murine models *in vivo* due to differences between media compositions in cell culture or fluid composition in the extracellular space. Previous studies demonstrating membrane destruction in bacteria have suspended Gr flakes with *E.Coli* (Tu et al., 2013) or suspended in agar/water and dropped onto a substrate surface and later recovered (Akhavan and Ghaderi, 2010). However, the presence of a protein corona (Cedervall et al., 2007) likely plays a role in mitigating these effects. Using A549 cells, multiple reports have demonstrated that the presence of FBS in normal culture media (Hu et al., 2011; Duan et al., 2015) or the addition of BSA (Li et al., 2014; Duan et al., 2015) result in lower cytotoxicity of GO flakes relative to serum-free media. Using a DPPC model membrane, molecular dynamics simulations revealed that BSA-coating of Gr reduces the total amount of lipid removal relative to bare Gr. The coating of Gr by proteins is governed by hydrophobic interactions, van der Waals forces, and π—π interactions (Chong et al., 2015). The composition of the protein corona may vary depending upon the method of introduction to the brain and the presence of any surface modifications to increase biocompatibility; this may also serve to explain the variation in effects seemingly exerted by Gr (Radic et al., 2013). As the adsorption of proteins to GO is strong and long-lasting, this may serve as one route for the low-cost modification of GO for drug delivery application or to achieve loss or gain of function cellular control in some manner (Belling et al., 2016).

Although many studies that attempt to evaluate the effects of Gr may lessen the extent to which a protein corona is involved by incubation in serum free-media (Zhang et al., 2010; Pampaloni et al., 2018), the relatively long exposure times used may still result in the coating of nanomaterial surfaces by excreted proteins in cell culture. Indeed, proposed mechanisms for the effects underlying chronic culture on graphene have suggested that Gr still plays a large and direct mediatory role, rather than an indirect through a protein corona (Kitko et al., 2018; Pampaloni et al., 2018). Understanding the role of cell secretion in mediating the toxicity of Gr-based materials remains an important line of future investigation.

Surface functionalization has also been shown to play an important role in the toxicity of Gr/GO. For example, PEGylation decreases the overall cytotoxicity of GO (Li et al., 2014). And bilayer Gr functionalized with carboxyl groups showed improved viability in kidney cells relative to pristine bilayer Gr at concentrations above ~5 mg/L (Sasidharan et al., 2011). Tu et al. (2013) modified GO with -OCH_3_, -NH_2_, or -PABS *via* PEGylated chains and cultured hippocampal neurons to 7 DIV to determine the effect of surface charge on neuronal viability and outgrowth. PEG-amine modified GO exhibited the most positive surface charge and the most neuronal outgrowth relative to other surface treatments or the native—COOH group (Tu et al., 2014), suggesting the importance of the surface in determining neuronal responses to GO. This is in line with what was observed for mouse hippocampal neurons on CVD-Gr substrates, where pristine Gr was shown to improve viability and connectivity up to 5 DIV, whereas disordered noncrystalline Gr did not result in any neuronal attachment (Veliev et al., 2016). Thus, the crystallinity of Gr is also an important consideration in evaluating neuronal responses. Given the overall inconsistency among assessments of nanotoxicity for Gr/GO, it is likely that surface charge plays a role. Given the number of fabrication methods, transfer processes, and application methods for these materials, comprehensive studies of toxicity should include measurement and reporting of relevant surface characteristics (Faria et al., 2018).

## Distribution and Trafficking of Gr/Go *in vivo*

Mass spectrometry is a common approach to determine the bio-distribution of nanomaterials. For Gr/GO, most distribution studies to date have focused on overall distribution following tail vein injection, with results indicating that very little trafficking to the brain will happen *via* this route. MALDI-TOF was used to determine that tail vein injection of GO results in very little accumulation in the brain after 24 h (Chen et al., 2015), in good agreement with what was observed using radiolabeled GO for similar time periods (Zhang et al., 2011). Tail vein studies performed using rGO indicate uptake in the brain within 15 min for tail vein injection, peaking around 3 h and decreasing by 7 days, which was corroborated by confocal microscopy (Mendonça et al., 2015). This is somewhat surprising given the relatively large-sized flakes used (~340 nm), suggesting that rGO may be able to cross the blood brain barrier and may be cleared through some as yet not well-understood mechanism.

For *in vivo* applications, further study is needed to characterize any potential brain region or cell-type specific effects. To date, most studies, such as detailed above, are performed *in vitro*. However, a few studies have characterized some of the effects of Gr/GO *in vivo*. Defteralí et al. (2016b) studied the effects of thermally reduced Gr on viability *via* stereotaxic injection into the mouse olfactory bulb. After 7 or 21 days, thermally reduced Gr had minimal effects on cell viability or number, and no significant increase in microglia number compared to injection only controls (Defteralí et al., 2016b). Tail vein injection of >250 mg/kg dextran-modified Gr did not lead to brain toxicity after 30 days (Kanakia et al., 2014). However, this is not the route by which Gr would encounter the brain in most intended applications, thus requiring further toxicity study *in vivo* *via* direct Gr/GO injection to brain regions of interest. Although this has not been characterized for Gr/GO-based materials, LDH activity has been shown to be differentially affected in a brain region-specific manner in multi-walled carbon nanotubes (MWNTs; Bussy et al., 2015). Thus, further study is warranted to determine if the toxicity is cell-type specific, as has been both suggested (Agarwal et al., 2010) and argued against (Ruiz et al., 2011).

## Graphene and Neurons *in vitro*

To date, even for *in vitro* systems, where there have been an array of studies using Gr/GO, questions remain as to toxicity. It is increasingly widely accepted that as a substrate for *in vitro* growth, Gr is a permissive surface both with and without the addition of extracellular matrix coatings (Table 1). Gr Flakes applied in culture have resulted in somewhat conflicting outcomes, but results generally support the idea that either or both high enough treatment dose or a long enough incubation time will result in cellular toxicity. Below these dosage or time thresholds, Gr flakes also have been studied for their ability to exert biological effects. Here, we move beyond toxicity to a discussion of hypotheses as to the causal underpinnings of biological changes reported on Gr/GO.

**Table 1:** Preclinical *in vitro* studies using Graphene (Gr) substrates.

Type of nanocarbon	Substrate coating	Culture system	Days in culture	Dendritic arbor on Gr	Synaptic properties	Reference
CVD Gr substrate	None	Rat hippocampal neurons	12–18	Slight increase	Synaptic strength increased	Kitko et al. (2018)
CVD Gr substrate	None	Rat hippocampal neurons	8–10	n/a	Synaptic strength increased	Pampaloni et al. (2018)
CVD Gr substrate	None	Mouse hippocampal neurons	5	Neurite length increased	n/a	Veliev et al. (2016)
CVD Gr substrate	PLL	Mouse hippocampal neurons	5	Neurite length increased	n/a	Veliev et al. (2016)
Gr on SiC substrate	None	Rat dorsal root ganglion neurons	2	Neurite length unchanged	n/a	Convertino et al. (2018)
CVD Gr substrate	None	Retinal ganglion cells	7	Unchanged number of neurite intersections	n/a	Fischer et al. (2018)
CVD Gr substrate	None	Rat hippocampal neurons	9	Neurite formation unchanged	n/a	Rastogi et al. (2017)
CVD Gr on sapphire substrate	None	Retinal ganglion cells	6	Neurite length unchanged	n/a	Bendali et al. (2013)
CVD Gr on sapphire substrate	Poly-D-lysine and laminin	Retinal ganglion cells	6	Neurite length increased	n/a	Bendali et al. (2013)
CVD Gr on tissue culture polystyrene	Poly-l-lysine	Mouse hippocampal neurons	2–7	Neuron length increased	n/a	Li et al. (2011)
CVD Gr on tissue culture polystyrene	Laminin	Neural stem cells	14	Neurite number unchanged	Increased spontaneous firing activity	Tang et al. (2013)
CVD Gr on tissue culture polystyrene	Poly-lysine	Rat hippocampal neurons	7–21	Increased dendritic complexity	Increased synaptic density, increased spontaneous firing activity	He et al. (2016)

*All substrates are on glass unless otherwise noted*.

Most early studies using Gr/GO substrates were conducted using a protein layer sandwiched between the substrate and neurons. For example, neural stem cells were grown on laminin-coated tissue culture polystyrene and soaked in tissue culture media overnight (Tang et al., 2013). Chronic culture resulted in increases in: Ca^2+^ transient frequency, both spontaneous EPSC amplitude and frequency, and miniature EPSC frequency. These cellular changes occurred without altering overall stem cell morphology. Later versions of similar studies using neural stems cells did not observe changes in firing frequency, although were in overall agreement with increased cell signaling, here realized as increases in the percentage of cells firing action potentials during both proliferation and differentiation stages (Guo et al., 2016) and an increase in the density of neurites. Longer-term culture of stem cells on Gr also acts in a supportive manner by increasing overall cell count on Gr after 1 month (Park et al., 2011). Again, surface properties of Gr/GO play an important role in stem cell proliferation and differentiation. For example, Defteralí et al. (2016a) used stem cells from adult mouse olfactory bulbs to test the effect of thermally reduced Gr and poly-vinylidene fluoride PVDF membranes loaded with multi-walled CNTs. Indeed, those nanomaterials significantly affect neurite branching and synapses during stem cell differentiation to neurons (Defteralí et al., 2016a,b). Similarly, changes during the differentiation of human induced pluripotent stem cells into multiple somatic cell lineages have been shown in graphene-treated samples (Yoo et al., 2014; Hu et al., 2015; Choi et al., 2016; Lee et al., 2016; Rodriguez-Losada et al., 2017; Wang et al., 2017; Nguyen et al., 2018; Sánchez-González et al., 2018; Saburi et al., 2019). The specific effects of Gr/GO seem to be variable even within stem cell type, but overall results collectively suggest that Gr holds promise as a scaffold material for regenerative medicine and stem cell-based technologies.

Observations regarding the formation of synaptic connections, a fundamental unit of neuronal signaling, largely indicate that Gr is both permissive and to some extent may also enhance synaptic transmission. E18 cortical (Keshavan et al., 2018) or E18 hippocampal (Lorenzoni et al., 2013) neurons cultured on poly-d-lysine coated Gr “stripes” have been used to investigate synaptogenesis, with results indicating that functional synaptic connections are formed on Gr substrates covered with an adhesion coating. P0-P1 rat (He et al., 2016) or mouse hippocampal neurons grown on Gr coated with poly-lysine and pre-incubated in culture media demonstrated longer and more branched dendrites after 7 DIV and increased synapse number after 21 DIV (He et al., 2016), suggesting that the enhancements observed on Gr may be the result of some sort of conserved mechanism. This collection of studies has used pre-incubation in media overnight in addition to ECM coating, as it was elsewhere demonstrated to mitigate the cytotoxicity of GO (Hu et al., 2011). The observed decrease in cytotoxicity after overnight media incubation calls raises questions regarding the complex interactions between nanomaterial surfaces, the adhesion layer, and growth factors, lipids, etcetera, that are contained in fetal serums.

Increasing numbers of studies are interfacing directly to Gr/GO, omitting the intermediate protein-coating layer. This omits confounding factors both of additional surface charges due to the complex nature of such coatings and the physical gap created between the biological material of interest and the substrate. For example, differences in surface charge been shown to alter neurite outgrowth on GO (Tu et al., 2014), with positively charged surfaces overall exhibiting increased neurite outgrowth at 7 DIV. More broadly, coatings like polylysine are polycationic polymers, increasing cell attachment and outgrowth—but it is unclear whether there would be coupling between the coating and Gr/GO, masking direct biological effects. And recent studies have begun to systematically investigate the different biological effects observed even between different classes of ECM substrate (Fischer et al., 2018), suggesting that a single underlying mechanistic explanation of the biological effects of Gr must fully account for the composition of the substrate. The biological compatibility of Gr/GO substrates also does not appear to be cell-type specific, as retinal ganglion cells (Bendali et al., 2013; Fischer et al., 2018), cortical neurons (Rauti et al., 2016), hippocampal neurons (Veliev et al., 2016; Kitko et al., 2018; Pampaloni et al., 2018), and recently dorsal root ganglion neurons (Convertino et al., 2018) have all been cultured on bare Gr. However, neurite outgrowth and total number were reduced in comparison to a bare glass control for retinal ganglion cells (Bendali et al., 2013). Interestingly, this same study shows no significant enhancement on coated Gr compared to coated glass in these same properties, in contrast to much of the published literature that utilizes a protein coating layer; and later studies have made somewhat different observations, where neurons were not viable on bare glass controls but formed synaptic connections on uncoated Gr (Veliev et al., 2016). This also included comparisons to protein-coated Gr, where enhancements in neuronal surface area relative to bare Gr were observed over the duration of the study (up to 5 DIV). Although this and other studies have suggested that synaptic connections are formed on Gr, later studies were left to determine synaptic function; and the effect of extracellular matrix addition on the ability to utilize the properties of Gr for neural recordings, for example, remains to be well-characterized.

The functional effects of Gr substrates have become an increasingly important area of study for neural interfacing applications. Multiple studies have now collectively suggested both that the frequency of neuronal firing is increased on bare Gr (Kitko et al., 2018; Pampaloni et al., 2018) and that synaptic strength is also increased (Rauti et al., 2016; Kitko et al., 2018), whereas high enough concentration treatment of neurons with GO flakes reduces EPSC frequency (Rauti et al., 2016). However, there are somewhat conflicting explanations for the mechanisms underlying this synaptic enhancement. It has been hypothesized (Kitko et al., 2018) that chronic growth on Gr results in increased neuronal membrane cholesterol. This increase in cholesterol, possibly through the extraction of cholesterol from a serum-containing media during the formation of a protein corona, is sufficient to explain the functional changes were observe on Gr. Specifically, Gr substrates result in an enlarged pool of synaptic vesicles and a higher vesicle release probability in neurons and potentiated Ca^2+^ release in 3T3 cells. More recently, an alternative explanation has been proposed for the increased firing frequency on graphene (Pampaloni et al., 2018). Pampaloni et al. (2018) hypothesize that K^+^ ions are depleted on bare graphene in the cleft between neurons and the substrate. This depletion of K^+^ ions alters neuronal signaling, increasing EPSC frequency and altering adapting vs. tonic firing phenotypes. Computational models support this hypothesis, with the caveat that protein deposition during chronic culture is not included.

**Figure 2:**
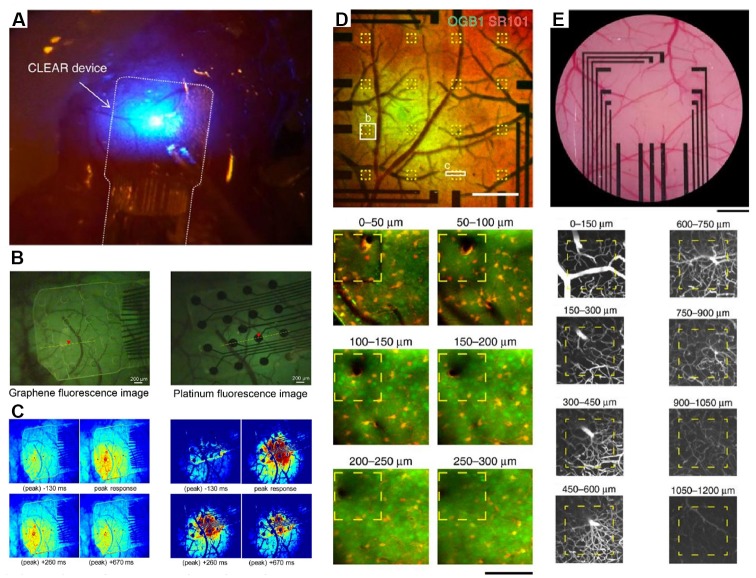
Integration of transparent electrodes with complementary optical techniques *in vivo*. **(A)** Gr-based electrodes implanted on a mouse cerebral cortex. An optical fiber is also shown and used to deliver optogenetic stimulation. Modified with permission from Park et al. (2014). **(B)** Gr-based electrodes implanted on a mouse cerebral cortex (left) or traditional platinum electrodes Howe et al. (2013) and peak calcium responses from the same regions **(C)**. Panels **(B,C)** are from Park et al. (2018). Copyright ACS Publication. **(D)** Two-photon calcium imaging to a depth of 300 μm through Gr-based electrodes and intravascular imaging **(E)** to a depth of 1,200 μm. Modified with permission from Thunemann et al. (2018).

Although optical technologies hold great promise for pre-clinical application, to date, electrodes remain the most widely used and sought-after new technology for neural interfacing for clinical applications (Figure 2). Traditionally, electrodes are designed based upon a silicon manufacturing workflow, allowing for larger scale production that is currently available for Gr- and any carbon-based nanomaterials. Advances based upon this technology that may hold promise for a path to clinical applicability were recently demonstrated (Park et al., 2018). Gr electrodes with over 90% transmittance have been fabricated on parylene-C or polyethylene terephthalate (PET) substrates, permitting simultaneous optogenetic stimulation (Park et al., 2014; Liu et al., 2018), optical coherence tomography (Park et al., 2014), deep vasculature (Thunemann et al., 2018) or Ca^2+^ imaging (Park et al., 2018; Thunemann et al., 2018) in areas where the surface would normally be blocked by the opacity of traditional recording materials.

An ongoing area of research will be to improve the electronic properties of Gr to better improve recording quality while, at the same time, maintaining transparency and flexibility, important for *in vivo* imaging applications. Recent data have suggested that nanoparticle doping may be an approach to meet all of these requirements. For example, platinum nanoparticles electrodeposited onto CVD Gr overcome the quantum capacitance limits of Gr electrodes alone and enable improved signal quality ECoG and EEG signals (Lu et al., 2018). These signals can be simultaneously acquired with signals from genetically encoded indicators such as GCaMP at depths up to 250 microns using two-photon excitation.

## Beyond the Murine Model: The Use of Graphene in Other Model Systems in Neuroscience

To date, most current understanding of the uses for and effects of Gr result from studies in murine models. Many of these studies are based on *in vitro* culture systems using neurons; future directions should include mechanistic characterization of the biological effects of Gr/GO *in vivo*. The large array of transgenic modifications possible in mice and increasingly rats have enabled the study of ever more complex behaviors in a cell-type specific manner. These new techniques have resulted in a concurrent rise in the number of studies using other model systems, which have provided fundamental insights into the function of molecules, cells, circuits, and brain regions. *Drosophila Melanogaster*, for example, is a widely used system for the study of synapses and synaptic proteins (Keshishian et al., 1996). And historically, the sea slug *Aplysia* provided some of the earliest causal insight into the mechanisms of plasticity in the brain (Kandel, 2009). We here provide a general introduction to model systems where Gr/GO have been used to date.

The worm *C. elegans* is a soil nematode with 302 neurons in its nervous system, whose connectome, a map of all neural connections, has been characterized (Varshney et al., 2011; Jarrell et al., 2012), leading to ongoing efforts to develop causal rules governing structure and function relationships during behavior. It lacks a blood-brain barrier, enabling screening of molecules by delivery routes not available to traditional murine models. Although Gr-based device interfaces have yet to be demonstrated in *C. elegans*, Li et al. (2017) have demonstrated that chronic exposure to Gr elicits toxicity effects that are dosage-dependent, cell-type specific, and dependent upon the type of Gr. Nematodes typically have a lifetime of ~2 weeks, and underwent a 6-day chronic exposure that resulted in Gr being distributed throughout the digestive system. 100 mg/L Gr flakes did not significantly alter the overall survival rate after 6 days, but the same concentration of GO flakes was largely lethal. Interestingly, >10 mg/L GO nanoparticles were shown to decrease expression levels of *dat-1* and *eat-4p*, fluorescent genes encoding dopaminergic and glutamatergic neurons respectively, without significant downregulation of *unc-47*, which encodes GABAergic neurons. This is in contrast to the behavior of graphite nanoplatelets, where no acute *in vivo* toxicity was observed at concentrations up to 250 mg/L (Zanni et al., 2012). This could be due in part to differences in surface energy between GO and graphite nanoplatelets, for example, hydrophilic vs. hydrophobic wettability properties, but further investigation is warranted with more standardized concentrations between materials. As computational models and subsequent experiments have suggested that similar concentrations of Gr flakes should be destructive to the membrane, regardless of cell type (Luan et al., 2017), it would thus be helpful for future studies interested in assessing toxicity to take into account the concentrations used in previous studies for better cross-comparison.

Zebrafish represent another interesting possibility for demonstrating the utility of G/GO to the study of the brain. A developed zebrafish has ~100,000 neurons, fewer than any murine model, while still preserving many basic electrical and chemical signaling processes. This, combined with optical transparency, confers many advantages for single-cell resolution studies involving a whole population of neurons rather than the subsets that are optically accessible by even the most recent imaging approaches, for example, light field microscopy in freely moving rodents (Skocek et al., 2018). The availability of detailed genomic information and the relatively high degree of homology to the human genome (~70%; Howe et al., 2013) confer distinct advantages to their use as a model system. The relative ease of breeding and maintaining zebrafish and their short lifespan are also advantageous relative to rodents or non-human primates for reducing cost in larger scale toxicology screens (Fako and Furgeson, 2009). Although a concentration of >120 mg/L single-walled carbon nanotubes (SWNTs) was shown to delay hatching in zebrafish (Cheng et al., 2007), primary sensory neurons were not developmentally affected. Further studies using Gr/GO to assess toxicology in zebrafish are necessary for comparison to the effects observed using SWNTs.

The use of Gr has largely been limited in non-human primate models, due in part to lingering questions as to toxicity. However, organizations such as the European Graphene Flagship have issued calls for the production of Gr electrode arrays for recording in both non-human primates and humans. This will necessitate further study of the biological effects of Gr and Gr-based devices. As new strategies are developed to handle and integrate the vast and wide-ranging data streams becoming more prevalent in modern neuroscience, non-traditional model systems will continue to play a role in helping to elucidate the brain. Thus, future research on the compatibility of Gr with other model organisms will help to clarify the utility of Gr to these systems.

## Prospects

The ultimate utility of Gr will be determined in part by its ability to be used in conjunction with the large array and wide variety of optical, chemical, and electrical tools commonly utilized in modern neuroscience. The ability to combine fast optical control that is tuned *via* real-time device-based feedback is another promising direction (Kim et al., 2017).

Gr was quickly recognized as a “wonder material” after its isolation, including recognition as the 2010 Nobel Prize in physics. The number of publications referencing graphene has jumped to several thousand per year, a quick rise from several hundred only 10 years ago. Spurred on by several large initiatives, including the NIH’s BRAIN and European Graphene Flagship, which represents the European Union’s largest single research initiative, new applications for Gr to broad areas of brain research should continue to be developed at a rapid pace. Yet there are still challenges remaining, including addressing the widespread utility of many Gr applications. The promises of nanomedicine are extensive; what remains to be seen is the extent to which new applications for nanomaterials deliver on the great promise so often espoused.

## Author Contributions

KK and QZ conceived and wrote the article.

## Conflict of Interest Statement

The authors declare that the research was conducted in the absence of any commercial or financial relationships that could be construed as a potential conflict of interest.
